# Green Tea Consumption and the COVID-19 Omicron Pandemic Era: Pharmacology and Epidemiology

**DOI:** 10.3390/life13030852

**Published:** 2023-03-22

**Authors:** Maksim Storozhuk, Siyun Lee, Jin I. Lee, Junsoo Park

**Affiliations:** 1 Bogomoletz Institute of Physiology, National Academy of Sciences of Ukraine, 01024 Kyiv, Ukraine; 2 Division of Biological Science and Technology, Yonsei University, Wonju 26493, Republic of Korea

**Keywords:** SARS-CoV-2, COVID-19, green tea, (-)-epigallocatechin-3-gallate (EGCG)

## Abstract

In spite of the development of numerous vaccines for the prevention of COVID-19 and the approval of several drugs for its treatment, there is still a great need for effective and inexpensive therapies against this disease. Previously, we showed that green tea and tea catechins interfere with coronavirus replication as well as coronavirus 3CL protease activity, and also showed lower COVID-19 morbidity and mortality in countries with higher green tea consumption. However, it is not clear whether green tea is still effective against the newer SARS-CoV-2 variants including omicron. It is also not known whether higher green tea consumption continues to contribute to lower COVID-19 morbidity and mortality now that vaccination rates in many countries are high. Here, we attempted to update the information regarding green tea in relation to COVID-19. Using pharmacological and ecological approaches, we found that EGCG as well as green tea inhibit the activity of the omicron variant 3CL protease efficiently, and there continues to be pronounced differences in COVID-19 morbidity and mortality between groups of countries with high and low green tea consumption as of December 6, 2022. These results collectively suggest that green tea continues to be effective against COVID-19 despite the new omicron variants and increased vaccination.

## 1. Introduction

The coronavirus pandemic caused by the SARS-CoV-2 virus led to hundreds of millions of infections and millions of deaths within the first year and is the deadliest pandemic of the 21st century [1,2]. At that time, without any vaccine available, many groups sought to identify effective and inexpensive therapies against COVID-19 [3,4,5,6,7,8,9]. Among these, there was mounting evidence suggesting the therapeutic potential of green tea catechins in the prevention/treatment of COVID-19. We and other groups identified green tea extract and the active ingredients of green tea, EGCG and theaflavin, as inhibitors of the SARS-CoV-2 3CL protease necessary for viral reproduction [10,11,12,13,14]. Furthermore, EGCG or green tea extract treatment reduced infection of SARS-CoV-2 as well as other human beta coronaviruses in human and primate cells [14,15,16,17,18], and reduced the viral load in the lung tissue of mice [17].

With a plethora of evidence that green tea extracts can counteract SARS-CoV-2 infection, the use of green tea as a therapy against COVID-19 is a distinct possibility. Still, it appears that there are only a few epidemiological studies assessing the therapeutic potential of green tea catechins: (i) an observational study reporting that people who consumed ≥4 cups/day of green tea had a lower, albeit statistically not significant, odds of SARS-CoV-2 infection [19]; and (ii) ecological studies reporting lower COVID-19 morbidity and mortality in countries with higher per capita green tea consumption [20,21].

However, the abovementioned ecological studies reflect the epidemiological situation globally before January 2021 [21]. Since then, two major changes in the coronavirus pandemic have occurred, namely a global mass vaccination campaign and the appearance of omicron SARS-CoV-2 variants [22,23]. How these two changes may alter the reported morbidity and mortality rates in countries with high green tea consumption is unknown, and whether green tea extract or EGCG is still effective in inhibiting SARS-CoV-2 omicron variants has yet to be tested.

A recent work, however, reports that EGCG from green tea effectively blocks infection of SARS-CoV-2 and variants of the virus [15]. The latter is in line with the observation that the neutralizing activity of concentrated green tea extract is independent of the strain of SARS-CoV-2 [24]. These studies point to the possibility that green tea extracts and EGCG may be just as effective in inhibiting SARS-CoV-2 omicron 3CL protease activity. The omicron variants contain one amino acid change (P132H) in the 3CL protease sequence, and Paxlovid, a coronavirus drug, is reported to also inhibit the 3CL protease variant (P132H) [25].

In this report, we sought to update the effect of green tea and green tea catechins upon SARS-CoV-2 and COVID-19. We used a two-prong approach utilizing both biochemical and epidemiological approaches. We generated the SARS-CoV-2 omicron 3CL protease (P132H) and examined whether green tea extract or EGCG are effective in inhibiting the activity of the omicron variant 3CL protease. We also asked whether we could still observe lower COVID-19 morbidity and mortality in countries with higher per capita green tea consumption in spite of growing vaccination rates and the appearance of new omicron variants of SARS-CoV-2.

## 2. Materials and Methods

### 2.1. Generation of SARS-CoV-2 3CL Protease Mutant (P132H)

The plasmid encoding His-tagged 3CL protease was described previously [11] and the point mutation of the 3CL protease (P132H) was generated using a Quickchange PCR mutagenesis with the enzyme nPFU forte (Enzynomics, Daejeon, Republic of Korea) using forward primer 5′-CCAATGTGCTATGAGGCACAATTTCACTATTAAGGG-3’ and reverse primer 5’-CCCTTAATAGTGAAATTGTGCCTCATAGCACATTGG-3’. The new plasmid containing the 3CL protease (P132H) was completely sequenced to verify the presence of the intended mutation only. His-tagged 3CL protease protein was prepared and purified as described previously [11]. The 3D structure of 3CL protease was generated using Pymol software (DeLano Scientific, Palo Alto, CA, USA).

### 2.2. Protease Assay for 3CL Protease Assay

A FRET-based protease assay was used to examine the protease activity of 3CL protease [26]. Briefly, Dabcyl-KTSAVLQSGFRKME-Edans was chemically synthesized (Anygen, Gwangju, Republic of Korea) and used for the SARS-CoV-2 3CL protease substrate. The 3CL protease activity was performed at 37 °C using 3CL protein and FRET peptide in the reaction buffer (20 mM Tris-HCl (pH 7.5), 200 mM NaCl, 5 mM EDTA, 5 mM DTT, and 1% DMSO) for 3 h. For the inhibition assay, the purified 3CL protease was incubated with EGCG for 1 h before the addition of substrate. The fluorescence was measured at 528 nm with excitation at 360 nm using a Synergy HTX multimode microplate reader (Biotek, Winooski, VT, USA). (−)-Epigallocatechin gallate (EGCG) (E4134, purity ≥ 95%) was purchased from Sigma-Aldrich (Saint Louis, MO, USA) and the green tea extract powder was provided by the AMOREPACIFIC R&I Center (Gyeonggi-do, Republic of Korea). Green tea extract contains epigallocatechin gallate (EGCG, 18.7 ± 1.2%), epigallocatechin (EGC, 11.2 ± 1.4%), epicatechin gallate (ECG, 3.8 ± 0.6%) and epicatechin (EC, 3.7 ± 0.6%) as active catechins (total catechin 37.4 ± 1.2%). To draw the inhibition curve, AAT Bioquest website program was used (https://www.aatbio.com/tools/ic50-calculator accessed on 1 December 2022).

### 2.3. Data Analysis Regarding COVID-19 Morbidity and Mortality

All data were obtained from open sources. Specifically, information about COVID-19 morbidity and mortality for a particular date was obtained from ‘Worldometers info. Coronavirus’. The information on ‘Worldometer’ is based on official daily reports and considered as a reliable source [27,28]. The methodological approach used in this report is similar to that described previously [21,29]. Nevertheless, some description of this approach is provided below with more details, and specific details of the current work are provided in Supplementary Materials. Briefly, information about COVID-19 morbidity (defined as total number of cases per million population) and mortality (defined as a total number of deaths per million population) for a specific date was directly obtained from ‘Worldometers info. Coronavirus’ (https://www.worldometers.info/coronavirus/ accessed on 6 December 2022). Analysis was restricted to 134 countries or territories (according to UN classification) with at least a population of 3 million. Twenty-one of these countries/territories, with estimated per capita green tea consumption above 150 g annually, were considered as a group with high consumption. Countries/territories with estimated per capita green tea consumption below 150 g were considered as a group with low consumption (see [21,29]. for details). Considering that COVID-19 morbidity and COVID-19 mortality do not follow a normal distribution (Urashima et al., 2020), a non-parametric statistic (Wilcoxon (Mann–Whitney U Test) for Unpaired Data) was used for comparisons.

In multiple linear regression analysis, the following factors as well as green tea consumption were included: population density, percentage of population aged above 65, percentage of urban population and Human Developmental Index (HDI). In a complementary analysis, an additional variable, namely vaccination rates, was added to the model. ‘KyPlot’ software was employed for statistical assessments.

## 3. Results

### 3.1. EGCG and Green Tea Extract Can Inhibit SARS-CoV-2 3CL Protease (P132H)

**Figure 1:**
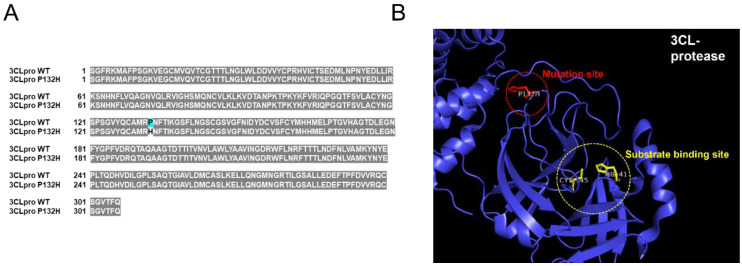
Sequence analysis of SARS-CoV-2 omicron 3CL protease. (**A**) SARS-CoV-2 omicron 3CL protease has one amino acid substitution (P132H). (**B**) Structural analysis of SARS-CoV-2 omicron 3CL protease indicates that the mutation site (P132H) is distant from the substrate binding site (PDB code 7T2T).

We previously showed using an in vitro assay that both EGCG and green tea extract can inhibit the 3CL protease activity of SARS-CoV-2 [10,11]. Since then, the SARS-CoV-2 omicron variants have replaced SARS-CoV-2 and become the dominant strains circulating globally causing the COVID-19 pandemic to extend into a third year in 2022. The SARS-CoV-2 omicron 3CL protease has been reported to contain one amino acid substitution (P132H) in its polypeptide sequence (Figure 1A). Although the location of the mutated sequence is quite distant from the substrate binding site (Figure 1B), whether such a mutation may alter 3CL protease activity and the ability of EGCG or green tea extracts to suppress protease activity is unknown.

**Figure 2:**
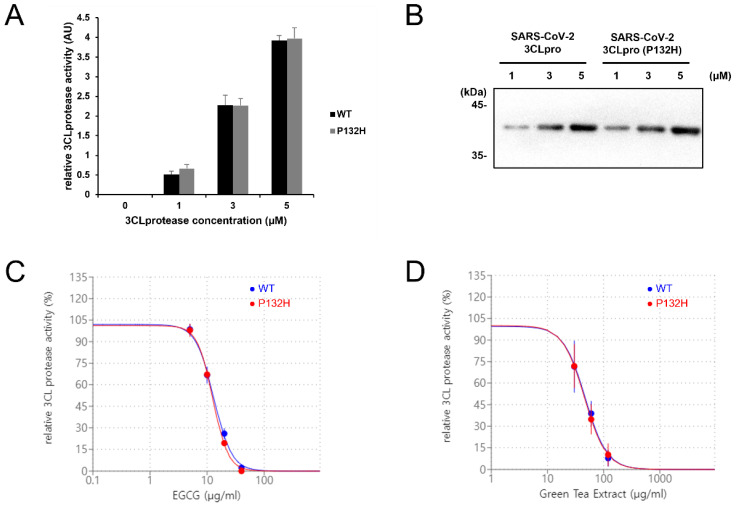
EGCG treatment inhibits the protease activity of SARS-CoV-2 omicron 3CL protease (P132H). (**A**) The protease activity of 3CL protease (P132H) is comparable to the protease activity of 3CL protease (WT). The graph shows the mean and standard deviation (n = 3). (**B**) The level of SARS-CoV-2 3CL protease was evaluated by Western blot. (**C**) EGCG treatment inhibits the protease activity of 3CL protease mutant (P132H) and wild type similarly. (**D**) Green tea extract treatment inhibits the protease activity of 3CL protease mutant (P132H).

To test whether EGCG or green tea extract can inhibit the protease activity of the 3CL protease variant (P132H), we produced SARS-CoV-2 3CL protease protein (P132H) and tested the enzyme activity using our in vitro assay. The purified 3CL protease protein (P132H) showed comparable activity to the original 3CL protease protein at all concentrations tested (Figure 2A,B), indicating that the mutation does not affect protease function. Next, we examined whether EGCG can inhibit protease activity by SARS-CoV-2 3 CL protease protein (P132H). EGCG treatment inhibits both original 3CL protease and 3CL protease (P132H) in a dose-dependent manner (Figure 2C). We also examined the inhibitory activity using green tea extract and showed that green tea extract treatment also effectively inhibits the protease activity of 3CL protease (P132H) in a dose-dependent manner similar to EGCG. (Figure 2D). Thus, SARS-CoV-2 3CL protease activity is conserved in SARS-CoV-2 omicron, and the ability of EGCG and green tea extracts to inhibit 3CL protease activity is not altered in SARS-CoV-2 omicron.

### 3.2. Higher Per Capita Green Tea Consumption Is Associated with Lower COVID-19 Morbidity and Mortality as of 6 December 2022

Consistent with the fact that green tea can inhibit SARS-CoV-2 3CL protease activity and decrease viral infectivity [10,11,14,15,18], we previously reported differences in COVID-19 morbidity and mortality between groups of countries/territories with higher and lower per capita green tea consumption [20,21]. The abovementioned results reflect the cumulative epidemiological situation in January 2021 or before, prior to the beginning of the mass vaccination campaign and appearance of the omicron SARS-CoV-2 variant. In light of our above findings that green tea extracts and EGCG can inhibit SARS-CoV-2 omicron 3CL protease activity (Figure 2), we asked whether these epidemiological differences remained in this current stage of the global pandemic.

Here, we mostly focused on the question of whether similar differences are still observed in spite of growing vaccination rates and the appearance of new variants of SARS-CoV-2. Therefore, using a similar approach, we primarily analyzed a recent 12-month period (6 December 2021–6 December 2022) separately (see Supplementary Materials for details). We found pronounced and statistically significant differences in COVID-19 mortality between groups of countries/territories with higher and lower green tea consumption (Table 1). The difference in COVID-19 mortality between the groups was still statistically significant in a subset of countries with a human development index (HDI) above 0.55 (Table S1). Moreover, in this restricted subset of countries, weak but statistically significant correlations between COVID-19 morbidity (or mortality) and per capita green tea consumption were observed using a multiple regression model accounting for several factors that have been reported previously as important confounders (population density, percentage of population aged above 65, percentage of urban population, HDI) [30] as well as vaccination rates [31]. These results are summarized in the Table S2 (a and b).

In addition, using a similar approach, we analyzed the cumulative COVID-19 morbidity and mortality for the entire COVID-19 pandemic period as of December 6, 2022. We obtained qualitatively similar results in this analysis (Tables S3–S5). Overall, both our biochemical studies using the SARS-CoV-2 omicron and epidemiological studies in the current stage of the global COVID-19 pandemic indicate that green tea remains a potential therapy against SARS-CoV-2 infection and COVID-19 disease.

## 4. Discussion

Since late 2019 when SARS-CoV-2 was first reported in China, many variants of the virus have appeared. Because many of the mutations of these variants occur at spike protein sequences [32], and these variants could potentially evade the human immune system [33], the SARS-CoV-2 3CL protease became a target of coronavirus drugs including Paxlovid [34]. Unlike the spike protein, the 3CL protease does not contain many mutations among the SARS-CoV-2 variants. The reason for this is likely because conservation of the protease’s important enzymatic function is necessary for the replication and success of the virus [35]. However, the SARS-CoV-2 omicron clade obtained a single amino acid mutation in the 3CL protease [25], although the location of this mutation is distant from the substrate binding site (Figure 1). Since green tea extracts and catechins showed inhibitory activity against 3CL protease [10,11,12,13,14], we decided to examine whether green tea extract and EGCG are also effective against the SARS-CoV-2 3CL protease mutant (P132H). We first showed that SARS-CoV-2 3CL protease mutant (P132H) activity is comparable to 3CL protease mutant; thus, the protease has retained its enzymatic function in the omicron strains. We also showed that EGCG and green tea extract is effective at inhibiting both 3CL proteases. Therefore, these results support that green tea or tea catechins are potentially effective against SARS-CoV-2 omicron variants. Since green tea and green tea catechins are known to inhibit SARS-CoV-2 infection, similar experiments can confirm whether they can also inhibit SARS-CoV-2 omicron infection.

Our in vitro experiments show that green tea catechins specifically inhibit 3CL protease enzyme function. Additionally, green tea has many positive effects on human health that can also contribute to fighting COVID-19. Green tea constituents are beneficial in relation to factors associated with higher COVID-19 mortality such as cholesterol levels [36], obesity [37,38], diabetes [39], uncontrolled immune activation [40], and cardiovascular disease [41]. Finally, green tea catechins can potentiate adaptive immunity [16] and can act as ionophores for zinc ions, the latter being considered as potentially beneficial in relation to COVID-19 [42].

Pronounced differences in COVID-19 morbidity and mortality between groups of countries/territories with higher and lower green tea consumption were found as of 6 December 2022 (Table S3). These results extend previous observations, reflecting the epidemiological situation in January 2021 [29] and before (September and November 2020) [20,21,29]. This consistency over a prolonged period may be an additional though indirect argument supporting the therapeutic potential of green tea catechins in the amelioration or treatment of COVID-19. These results are in line with the rapidly growing evidence obtained from other studies in a recent review [43]. Additionally, the selective analysis of the epidemiological situation during the most recent one-year period suggests that green tea catechins may be effective even with growing vaccination rates and against new variants of SARS-CoV-2 including omicron. This is consistent with our pharmacological evidence obtained in the current study (Figure 2).

Although ecological studies, taken alone, could not confirm a causal relation, these studies are still considered as useful and widely used in the field [27,30]. Limitations and potential concerns relevant to our current ecological results have been discussed in more detail previously [21,29], and briefly outlined below. Indeed, there are many factors that can differentially affect COVID-19 morbidity and mortality in distinct countries (e.g., the percentage of older population; administrative strategies to prevent transmission; condition-specific mortality risks; HDI). On the other hand, since numerous countries from all over the world were considered, it does not seem likely that these factors can systematically or strongly bias the results presented here. Furthermore, confounding factors reported as the most strong and consistent (HDI, percentage of older population) as well as other factors were included in our linear regression model. In this study, in addition to these factors, we address the potential concern of how differences in vaccination rates may bias our current results. Nonetheless, statistically significant correlations between COVID-19 morbidity and mortality and per capita green tea consumption were still observed in a linear regression model that included vaccination rates (Table S5). A separate though related question is whether the efficacy of green tea catechins in lowering COVID-19 morbidity and mortality remains consistent when vaccination rates in a population are increased. A direct answer to this question cannot be obtained using our ecological approach alone. However, a preliminary clue can be derived from our data: since the strength of correlations we consider here seems to be weaker during the recent 1-year period (Table S2) compared to cumulative data since the beginning of the epidemic (Table S5), a decrease in efficacy cannot necessarily be excluded. Given that this is the case rather than due to the appearance of new variants of SARS-CoV-2, one possibility is that green tea catechins can provide a non-additive action on the immune system consistent with their role in potentiating adaptive immunity [16]. Taken together, if the efficacy of green tea or green tea catechins (e.g., EGCG) can be confirmed in observational studies and clinical trials in combination with the results shown in this study, green tea or green tea catechins can be used as an inexpensive method to prevent or relieve COVID-19 diseases.

## Figures and Tables

**Table 1:** Lower COVID-19 mortality in the group of countries with higher per capita green tea consumption (the increases in COVID-19 morbidity and mortality during a recent one-year period).

	Group 1 (Countries/Territories with ‘High’ Green Tea Consumption) N = 21	Group 2 (Countries/Territories with ‘Low’ or Undetermined Green Tea Consumption) N = 113	Group 3 (Countries/Territories with ‘Low’ Green Tea Consumption) N = 82
COVID-19 Morbidity	8439 (841–103,023)	22,091 (1293–119,227)	45,228 (4136–168,640)
COVID-19 Mortality	32 (6–169)	159 (9–511)	254 * (21–595)

Values (per one million of population) are: median and interquartile range (IQR). * (*p* < 0.05) denotes significance level of difference compared to Group 1 (Wilcoxon (Mann–Whitney U Test) for Unpaired Data). One year period, 6 December 2021–6 December 2022.

## Data Availability

Publicly available datasets were analyzed in this study. These data can be found here: https://www.worldometers.info/coronavirus/ accessed on 6 December 2022.

## Supplementary Materials

The following supporting information can be downloaded at: https://www.mdpi.com/article/10.3390/life13030852/s1, Details regarding epidemiological approach used in this study. Table S1: The difference in COVID-19 mortality between groups with higher and lower per capita green tea consumption is still statistically significant in a subset of counties with a human development index (HDI) above 0.55; Table S2: (A) Higher per capita green tea consumption is associated with lower COVID-19 morbidity in the model accounting for several confounding factors including vaccination (increases in COVID-19 morbidity and mortality during recent one year period, (B) Higher per capita green tea consumption is associated with lower COVID-19 mortality in the model accounting for several confounding factors including vaccination (increases in COVID-19 morbidity and mortality during recent one year period); Table S3: Lower COVID-19 morbidity and mortality in the group of countries with higher per capita green tea consumption (cumulative data since the beginning of the epidemic to 6 December 2022); Table S4: COVID-19 morbidity and mortality in groups of countries with higher and lower per/capita green tea consumption in a subset of countries with HDI above 0.55 (cumulative data since the beginning of the epidemic to 6 December 202); Table S5: (A) Higher per capita green tea consumption is associated with lower COVID-19 morbidity in the model accounting for several confounding factors (cumulative data since the beginning of the epidemic to 6 December 2022), (B) Higher per capita green tea consumption is associated with lower COVID-19 mortality in the model accounting for several confounding factors: cumulative data since the beginning of the epidemic to 6 December 2022.
