# Supplementary material for: Green Tea Consumption and the COVID-19 Omicron Pandemic Era: Pharmacology and Epidemiology
Source: Life (Basel). 2023 Mar 22;13(3):852. doi: 10.3390/life13030852 (PMC10054848; doi:10.3390/life13030852)
Supplement: Supplementary file 1 [file life-13-00852-s001.zip › life-2216612-supplementary.pdf]

This file contains: Details regarding epidemiological approach used in this study;

Supplementary Tables S1-S5

Details regarding epidemiological approach

Data regarding COVID-19 morbidity and mortality

As already mentioned, the methodological approach used in this report is similar to that described previously [1,2]. Information about COVID-19 morbidity (defined as total number of cases per million population) and mortality (defined as total number of deaths per million population) for a specific date was directly obtained from 'Worldometers info. Coronavirus.' The information on 'Worldometer' is based on official daily reports and considered reliable [3,4]. Analysis was restricted to countries or territories (according UN classification) with at least a population of 3 million listed on 'Worldometers info.' Twenty-one of these countries/territories, with estimated *per capita* green tea consumption above 150 g annually, were considered as a group with high consumption. Countries/territories with estimated *per capita* green tea consumption below 150 g were considered as a group with low consumption (see [1,2] for details).

In the first part of analysis a recent one-year period was analyzed separately. For this period increases in COVID-19 morbidity and mortality were calculated. Specifically, based on data provided on 'Worldometer' as of December 6, 2022 and December 6, 2021, differences in total number of cases/deaths were calculated first, then these values were divided by country population (in millions). This specific period for analysis was chosen because: 1) it is reasonably

long; 2) corresponds to a calendar year to minimize seasonal variations in morbidity and mortality and reduce potential bias; 3) started some time after initiation of vaccination (thus vaccination rates during this period were not too low). Availability of archived data regarding the cases/deaths was an additional reason for choosing this specific period.

In the second part of analysis *cumulative* data since the beginning of the epidemic to December 6, 2022 were considered. Thus, periods before and after the beginning of mass vaccination against SARS-CoV2 were analyzed together. However, unlike in December 2022, when data for 135 countries/territories with population of at least 3 million were listed on Worldometer, in December 2021 the number of corresponding countries/territories was 134. (Information about DPRK was not available as of December 6, 2021 and before). Therefore, the analysis of differences was restricted to 134 countries/territories (thus **n** for Group 2 in **Table 1** and **Table S3** are different). It should be also mentioned that DPRK was included in analysis of cumulative morbidity and mortality.

Statistical analysis

Since the variables of COVID-19 morbidity and COVID-19 mortality do not have a normal distribution [3], a non–parametric statistic was primarily employed for the analysis as suggested earlier [3]. For multiple linear regression analysis, an approach similar to that previously reported [3] was used. In this analysis, morbidity and mortality per million of the population were transformed into common logarithm (log10) to adjust for normality of the distribution, as suggested previously [3]. For the same reason *per capita* green tea consumption was also transformed into common logarithm (log10), which notably decreased skewness of the

distribution of this variable. Logarithmic dependence between dose and effect, usually observed in pharmacological studies, was an additional argument to use this transformed variable in multiple linear regression models. Other factors included in multiple linear regression analysis, were population density, percentage of population aged above 65, percentage of urban population, and Human Developmental Index (HDI), which is based on access to health and education services and income (UN, 2019). In the analysis of the recent one-year period, vaccination rates were also included in the linear regression model. The values were retrieved from Online Resource OurWorldInData.org (https://ourworldindata.org/coronavirus). Specifically, values as from June 6, 2022 (i.e. corresponding to the middle of the period analyzed in this study) were used for analysis.

'KyPlot' software was employed for statistical assessments. Values provided in **Tables S2** and **S3** are main values obtained in the multiple regression model copy-pasted from 'KyPlot'. Values in **Table 1**, **S1**, **S3** and **S4** are rounded to the nearest integer.

Supplementary Tables S1-S5

Table S1. The difference in COVID-19 mortality between groups with higher and lower per capita green tea consumption is still statistically significant in a subset of counties with a human development index (HDI) above 0.55

.

	Group 1 (countries/territories with 'high' green tea consumption) N=15	Group 3 (countries/territories with 'low' green tea consumption) N=69
COVID-19 morbidity	30001 (4986-183964)	70743 (21910-205834)
COVID-19 mortality	58 (18-288)	335* (73-649)

Values (per one million of population) are: *increases* in COVID-19 morbidity and mortality during recent one year period (December 6, 2021 –December 6, 2022). Values are presented as median and interquartile range (IQR). * ($P<0.05$) denotes significance level of difference compared to Group 1 (Wilcoxon (Mann-Whitney U Test) for Unpaired Data).

Table S2.

A. Higher *per capita* green tea consumption is associated with lower COVID-19 morbidity in the model accounting for several confounding factors including vaccination (*increases* in COVID-19 morbidity and mortality during recent one year period)

Regression Coefficient

	Estimate	SE	t(cal)		P(T<=t(cal))	Lower 95%	Upper 95%	VIF (Variance Inflation Factor)
b0	0.701524	0.549876	1.275787	N.S. (P>0.05)	0.205866	-0.393419	1.796468	
b1:X1	-0.152602	0.074278	-2.054477	* (P<=0.05)	0.043322	-0.300508	-0.004696	1.140704
b2:X2	0.000013	0.000050	0.265246	N.S. (P>0.05)	0.791529	-0.000086	0.000112	1.110387
b3:X3	0.035359	0.012968	2.726524	** (P<=0.01)	0.007922	0.009535	0.061182	2.697964
b4:X4	0.003803	0.004629	0.821543	N.S. (P>0.05)	0.413873	-0.005415	0.013020	2.251812
b5:X5	4.105162	1.134907	3.617179	*** (P<=0.001)	0.000530	1.845273	6.365050	5.506337
b6:X6	0.003173	0.003169	1.001389	N.S. (P>0.05)	0.319775	-0.003137	0.009483	1.619164

Standard Regression Coefficient

	Estimate	SE		
b1:X1	-0.137078	0.066722	X1	Log (*per capita* green tea consumption)
b2:X2	0.017461	0.065829	X2	population density
b3:X3	0.279774	0.102612	X3	%population aged above 65
b4:X4	0.077015	0.093745	X4	urban population % of total
b5:X5	0.530251	0.146592	X5	HDI
b6:X6	0.079603	0.079492	X6	% of fully vaccinated
			Y	Log(cases/million)

B. Higher *per capita* green tea consumption is associated with lower COVID-19 mortality in the model accounting for several confounding factors including vaccination (*increases* in COVID-19 morbidity and mortality during recent one year period)

Regression Coefficient

	Estimate	SE	t(cal)		P(T<=t(cal))	Lower 95%	Upper 95%	VIF (Variance Inflation Factor)
b0	-0.325002	0.542582	-0.598991	N.S. (P>0.05)	0.550937	-1.40542	0.755417	
b1:X1	-0.248357	0.073293	-3.388574	** (P<=0.01)	0.00111	-0.3943	-0.10241	1.140704
b2:X2	6.12E-06	4.91E-05	0.1244513	N.S. (P>0.05)	0.901282	-9.17E-05	0.000104	1.110387
b3:X3	0.053766	0.012796	4.201634	*** (P<=0.001)	7.07E-05	0.028285	0.079247	2.697964
b4:X4	-0.000239	0.004568	-0.05238	N.S. (P>0.05)	0.958364	-0.00933	0.008856	2.251812
b5:X5	3.17E+00	1.12E+00	2.8282743	** (P<=0.01)	0.005963	0.937339	5.40E+00	5.506337
b6:X6	-0.003618	0.003127	-1.157232	N.S. (P>0.05)	0.250755	-0.00984	0.002608	1.619164

Standard Regression Coefficient

	Estimate	SE		
b1:X1	-0.249174	0.073534	X1	Log (*per capita* green tea consumption)
b2:X2	0.0090289	0.07255	X2	population density
b3:X3	0.4751556	0.113088	X3	%population aged above 65
b4:X4	-0.005411	0.103316	X4	urban population % of total
b5:X5	0.456933	0.161559	X5	HDI
b6:X6	-0.101383	0.087608	X6	% of fully vaccinated
			Y	Log(deaths/million)

One year period - December 6, 2021 –December 6, 2022. n=84 (countries with HDI above 0.55)

Table S3. Lower COVID-19 morbidity and mortality in the group of countries with higher *per capita* green tea consumption (*cumulative* data since the beginning of the epidemic to December 6, 2022)

	Group 1 (countries/territories with 'high' green tea consumption) N=21	Group 2 (countries/territories with 'low' or undetermined green tea consumption) N=114	Group 3 (countries/territories with 'low' green tea consumption) N=82	World[1]
COVID-19 morbidity	23940 (5034-116400)	73871 (N.S P=0.22) (5053-226594)	122969* (16901-283640)	115678
COVID-19 mortality	233 (48-608)	734* (101-2134)	1219** (213-2515)	793

Values (per one million of population) are: median and interquartile range (IQR). * (P<0.05) and ** (P<0.01) denote significance level of difference compared to Group 1 (Wilcoxon (Mann-Whitney U Test) for Unpaired Data). Raw data for individual countries are from 'Worldometer' (Worldometers info. Coronavirus update. Available at: https://www worldometers info/coronavirus/) as provided on December 6, 2022. World[1] – refers to top 212 countries/territories (ranked by population) affected by COVID-19 according to information from 'Worldometer' as provided on December 6, 2022.

Table S4. COVID-19 morbidity and mortality in groups of countries with higher and lower per/capita green tea consumption in a subset of countries with HDI above 0.55 (*cumulative* data since the beginning of the epidemic to December 6, 2022)

	Group 1 (countries/territories with 'high' green tea consumption) N=15	Group 3 (countries/territories with 'low' green tea consumption) N=69
COVID-19 morbidity	95219 (15567-243119)	164548 (58879-309132)
COVID-19 mortality	436 (193-780)	1632** (624-2691)

Values (per one million of population) are: median and interquartile range (IQR). ** (P<0.01) denotes significance level of difference compared to Group 1 (Wilcoxon (Mann-Whitney U Test) for Unpaired Data). Raw data for individual countries are from 'Worldometer' (Worldometers info. Coronavirus update. Available at: https://www worldometers info/coronavirus/) as provided on December 6, 2022.

Table S5.

A. Higher *per capita* green tea consumption is associated with lower COVID-19 morbidity in the model accounting for several confounding factors (*cumulative* data since the beginning of the epidemic to December 6, 2022).

Regression Coefficient

	Estimate	SE	t(cal)		P(T<=t(cal))	Lower 95%	Upper 95%	VIF (Variance Inflation Factor)
b0	1.75667273	0.45692893	3.84452066	*** (P<=0.001)	0.00024503	0.8469971	2.66634836	
b1:X1	-<br>0.15502589	0.06355693	-2.4391658	* (P<=0.05)	0.01699265	-<br>0.28155802	-0.02849376	1.13691328
b2:X2	-7.74E-06	4.26E-05	-<br>0.18194659	N.S. (P>0.05)	0.85609666	-9.25E-05	7.70E-05	1.10371991
b3:X3	0.02153925	0.0109897	1.95994808	N.S. (P>0.05)	0.05357357	-<br>0.00033957	0.04341807	2.63741114
b4:X4	0.00361095	0.00396505	0.9106929	N.S. (P>0.05)	0.36526301	-<br>0.00428287	0.01150477	2.24909949
b5:X5	3.70749822	0.89671163	4.13454905	*** (P<=0.001)	8.89E-05	1.9222825	5.49271394	4.6794592

Standard Regression Coefficient

	Estimate	SE		
b1:X1	-0.17514798	0.07180651	X1	Log (per capita green tea consumption)
b2:X2	-0.01287281	0.07075051	X2	population density
b3:X3	0.214355	0.10936769	X3	%population aged above 65
b4:X4	0.0919764	0.10099607	X4	urban population % of total
b5:X5	0.60231838	0.14567934	X5	HDI
			Y	Log(cases/million)

B. Higher *per capita* green tea consumption is associated with lower COVID-19 mortality in the model accounting for several confounding factors: *cumulative* data since the beginning of the epidemic to December 6, 2022.

Regression Coefficient

	Estimate	SE	t(cal)		P(T<=t(cal))	Lower 95%	Upper 95%	VIF (Variance Inflation Factor)
b0	1.259738	0.493840	2.550902	* (P<=0.05)	0.012701	0.276578	2.242899	
b1:X1	-0.263216	0.068691	-3.831872	*** (P<=0.001)	0.000256	-<br>0.399969	-0.126462	1.136913
b2:X2	-0.000069	0.000046	-1.497443	N.S. (P>0.05)	0.138314	-<br>0.000160	0.000023	1.103720
b3:X3	0.029710	0.011877	2.501380	* (P<=0.05)	0.014466	0.006064	0.053356	2.637411
b4:X4	0.003356	0.004285	0.783047	N.S. (P>0.05)	0.435970	-<br>0.005176	0.011887	2.249099
b5:X5	1.897153	0.969150	1.957544	N.S. (P>0.05)	0.053860	-<br>0.032276	3.826581	4.679459

Standard Regression Coefficient

	Estimate	SE		
b1:X1	-0.336295	0.087763	X1	Log (per capita green tea consumption)
b2:X2	-0.129487	0.086472	X2	population density
b3:X3	0.334360	0.133670	X3	%population aged above 65
b4:X4	0.096658	0.123438	X4	urban population % of total
b5:X5	0.348542	0.178051	X5	HDI
			Y	Log(deaths/million)

References for Supplementary

1. Storozhuk, M. Green Tea Catechins Against COVID-19: Lower COVID-19 Morbidity and Mortality in Countries with Higher per capita Green Tea Consumption. *Coronaviruses* **2022**, *3*, 57-64, doi:10.2174/2666796703666220124103039.
2. Storozhuk, M. COVID-19 and per capita green tea consumption: update. *medRxiv* **2022**, 2022.2006.2006.22276060, doi:10.1101/2022.06.06.22276060.
3. Urashima, M.; Otani, K.; Hasegawa, Y.; Akutsu, T. BCG Vaccination and Mortality of COVID-19 across 173 Countries: An Ecological Study. *Int J Environ Res Public Health* **2020**, *17*, doi:10.3390/ijerph17155589.
4. Klinger, D.; Blass, I.; Rappoport, N.; Linial, M. Significantly Improved COVID-19 Outcomes in Countries with Higher BCG Vaccination Coverage: A Multivariable Analysis. *Vaccines (Basel)* **2020**, *8*, doi:10.3390/vaccines8030378.
